# Eagle Syndrome

**DOI:** 10.5811/cpcem.2018.8.39503

**Published:** 2018-09-05

**Authors:** Elisha Bremmer, Shane Sergent, John Ashurst

**Affiliations:** Kingman Regional Medical Center, Department of Emergency Medicine, Kingman, Arizona

## CASE PRESENTATION

A 30-year-old male presented to the emergency department with intermittent neck pain, dysarthria, right facial droop, right-sided facial paresthesias and right upper extremity weakness for several days prior. Past medical history was significant for hypertension. Neurologic exam revealed a National Institutes of Health Stroke Scale (NIHSS) score of three secondary to dysarthria, right facial paralysis, and mild right upper extremity hemiparesis. Noncontrast brain computed tomography (CT) showed no evidence of hemorrhage, mass lesion, or acute infarction. CT angiography (CTA) head and neck with three-dimensional rendering demonstrated a large left styloid process and partially calcified stylohyoid ligament and large completely calcified right stylohyoid ligament consistent with Eagle syndrome (Image). The left cervical internal carotid artery also had severe focal dissection and 99% narrowing. The right cervical internal carotid artery had mild narrowing and intimal irregularity consistent with carotid dissection. Magnetic resonance imaging of the brain demonstrated scattered infarcts predominantly in a band-like pattern within the deep white matter of the left frontal lobe. The patient was admitted to the hospital after neurology consultation and started on enoxaparin. However, he decided to forego further definitive surgical management of his Eagle syndrome.

## DIAGNOSIS

Eagle syndrome is relatively uncommon with an incidence of abnormal stylohyoid length being 4% to 7.3%.^1^ Classic Eagle syndrome is described as post-tonsillectomy pain, dysphagia and a foreign-body sensation, while the less-common version is related to an elongated styloid which can compress the carotid artery.^1^ Transient ischemic attacks, cerebral vascular accidents and carotid artery dissections are all related to the second form of Eagle syndrome and were all seen in our patient.^1^ CT represents the gold standard for diagnosis, and CT angiography can provide the clinician with further data in regard to the carotid artery. Management can be anything from conservative therapy through definitive surgical removal of the styloid process.^1^

**CPC-EM Capsule**

What do we already know about this clinical entity?

Eagle syndrome can present as an elongated styloid that can compress the carotid artery. If the latter occurs, patients are at risk of stroke and carotid artery dissection.

What is the major impact of the image(s)?

The image depicts a classic example of Eagle syndrome in which the patient suffered from stroke-like symptoms with associated carotid artery dissection.

How might this improve emergency medicine practice?

Emergency physicians should be aware that there are numerous rare causes of stroke-like symptoms, especially in the younger population.

Documented patient informed consent and/or Institutional Review Board approval has been obtained and filed for publication of this case report.

## Figures and Tables

**Image:**
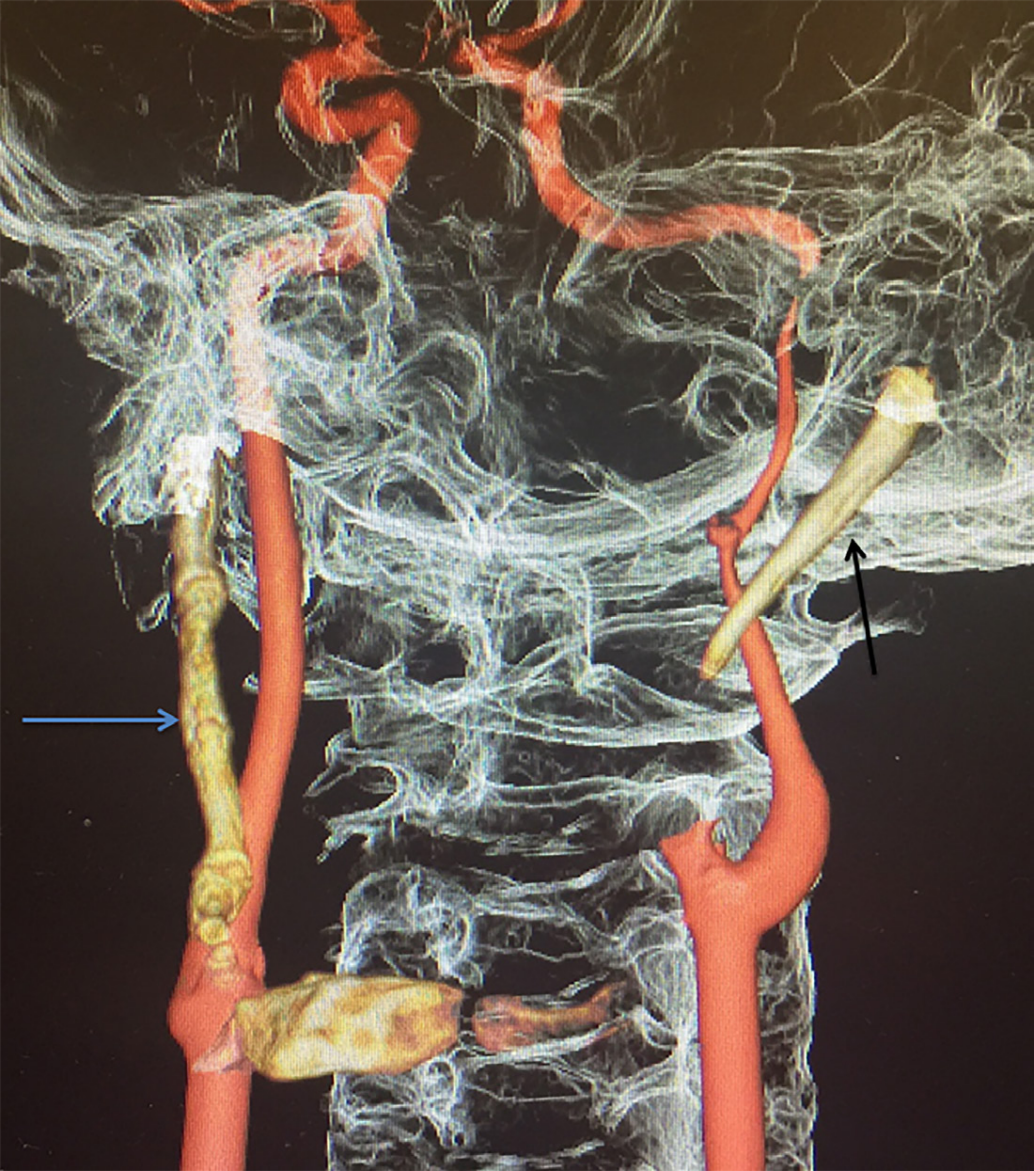
Computed tomography angiography with three-dimensional rendering showing large left styloid process (black arrow) and large completely calcified right stylohyoid ligament (blue arrow).
